# Shoreline classification maps and ground truth data for the Neuse River Estuary, North Carolina

**DOI:** 10.1038/s41597-024-02954-5

**Published:** 2024-01-22

**Authors:** Hannah Sirianni, Jessica Richter, Matthew J. Sirianni, Sarah Pettyjohn

**Affiliations:** 1 Department of Geography, Planning and Environment, East Carolina University, Greenville, USA; 2 Department of Geological Sciences, East Carolina University, Greenville, USA

**Keywords:** Natural hazards, Conservation biology, Geography

## Abstract

Estuaries provide essential ecosystem services and economic value but are facing widespread degradation due to changing anthropogenic and climatic factors. In North Carolina, coastal structures, like bulkheads and riprap, are widely used by property owners throughout the Albemarle-Pamlico estuary to stop erosion and reclaim lost land following storm events. While coastal development is tightly governed, limited historical and no up-to-date data report on the spatial distribution of coastal structures throughout the Albemarle-Pamlico estuary. Here we describe the development of a dataset that classifies and catalogues 67 km of shoreline type along the Neuse River Estuary (NRE), a large tributary of the Albemarle-Pamlico. We used available LiDAR digital elevation models (DEMs), aerial imagery, and a ground truthing field campaign to determine shoreline type present along the NRE as of 2020. We validated these results using an intensive manual editing procedure that comparatively examines DEMs, LiDAR derived slope, aerial imagery, and ground truth photography of the shoreline. This dataset is available for public download.

## Background & Summary

Estuarine environments are complex coastal systems at the interface between rivers and oceans. Here, hydraulic energy manipulates the land through erosion and deposition, and freshwater and seawater interact to control critical, yet delicate biogeochemical functions and food webs^1–3^. Despite estuaries providing essential ecosystem services^1^ and economic value^4^, centuries of human activities have resulted in widespread degradation and biodiversity loss that not only undermines ecological resilience but ultimately human community resilience as well^1,4,5^.

Recognizing the importance of protecting estuaries, the U.S. Congress enacted the Coastal Zone Management Act of 1972 (16 U.S.C. 1451–1464), encouraging states to regulate the management and development of coastal areas. This legislation holds particular significance for states like North Carolina, which has the second-largest estuarine complex in the US, the Albemarle-Pamlico estuary. The Albemarle-Pamlico estuary stands as one of the most biologically productive regions in the country but, like many other estuaries, faces mounting challenges due anthropogenic factors^6^, rising sea levels^7^, and increased storm intensity and frequency^7,8^.

In response to these challenges, the North Carolina General Assembly enacted the Coastal Area Management Act of 1974 (1973, c. 1284, s. 1; 1975, c. 452, s. 5; 1981, c. 932, s. 2.1.) which aimed to provide a program for the protection, preservation, development, and management of North Carolina’s coastal resources. The Coastal Area Management Act of 1974 also established the Coastal Resources Commission (CRC), which (1) designates areas of environmental concern, (2) adopts rules and policies for coastal development in those areas, and (3) certifies local land use plans. Thus, the CRC governs coastal development which includes, among other things, the construction of erosion control structures like bulkheads, riprap, and other shoreline stabilization methods.

Permanent erosion control structures are used along North Carolina’s estuarine shorelines to (1) halt or slow coastal erosion, (2) reclaim lost land after storm events, and (3) protect coastal habitats^9^. Given the widespread usage of erosion control structures by property owners, the North Carolina Division of Coastal Management, which carries out the Coastal Area Management Act of 1974 using the rules and policies of the CRC, has interest in understanding the statewide usage of these structures and how these structures may impact ecosystem function, water quality, fisheries, wetland habitat, and other natural resources^9^.

In large estuarine systems, such as the Albemarle-Pamlico estuary, digitizing the physical interface between the land and water and segmenting the shoreline into unique classifications is challenging due to several factors, including dense vegetation, structures, and shadows^10,11^. While recent advancements in digital image processing techniques such as object-based image analysis and machine-learning classification can make the shoreline classification more efficient while minimizing cost, their use in identifying features of obscured shorelines can be complicated^12^.

Starting in 2007, a project to develop a protocol for manually classifying the estuarine shoreline^9^ and assess the state of North Carolina’s estuarine shoreline^10^ was carried out. Since its completion in 2010, and to the authors’ knowledge, no additional high-resolution shoreline mapping efforts have been conducted. Therefore, this project aimed to provide updated digital representations of the shoreline that can aid in (1) evaluating rules and policies within areas of environmental concern, (2) identify specific locations for restoring ecosystem function, and (3) quantify shoreline erosion and the effects of erosion control structures.

**Fig. 1:**
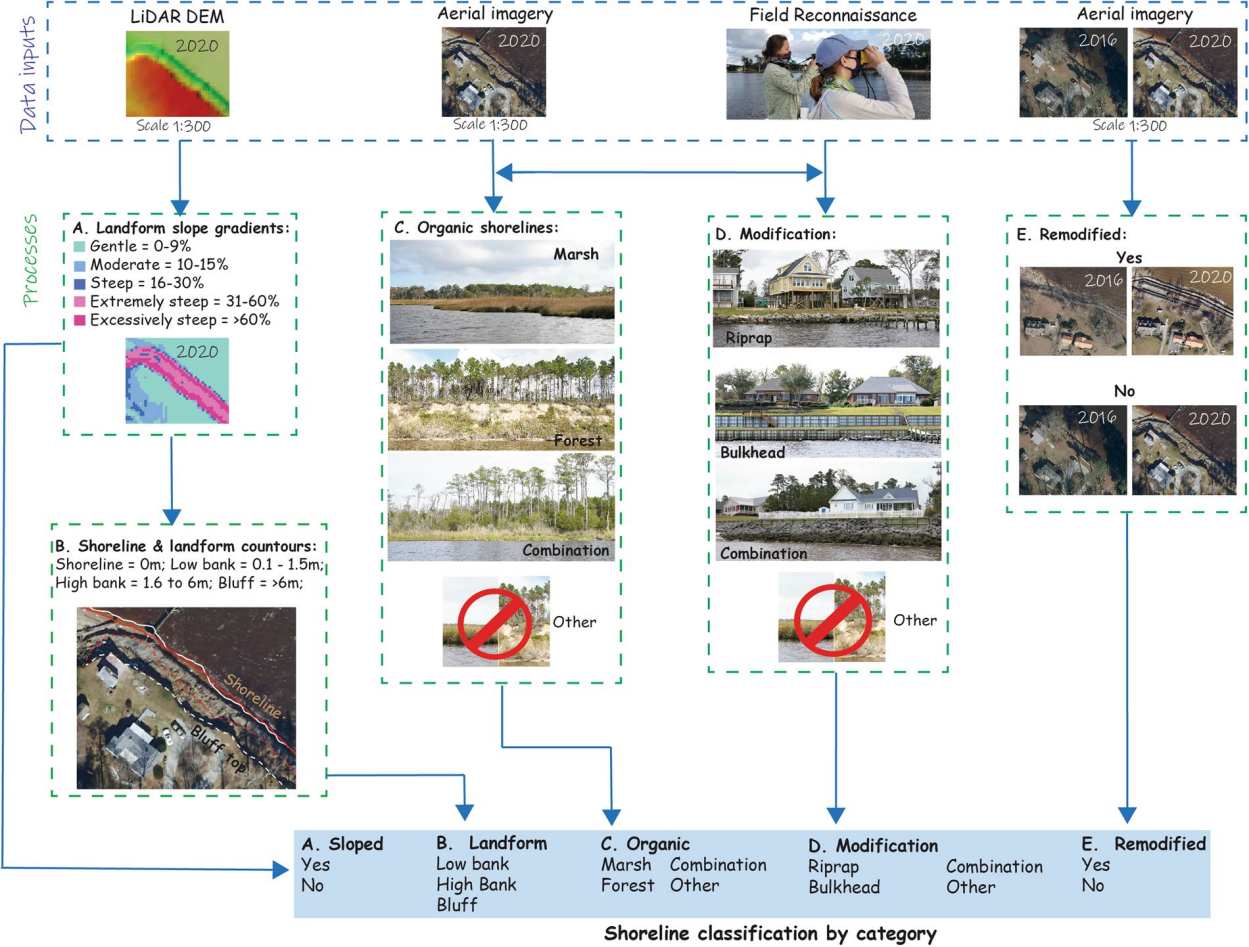
A schematic overview of the multi-step process used to define and categorize the shoreline objectively. The blue dashed box represents data inputs, while the green dashed boxes denote processes, such as creating a sloped surface from the LiDAR DEM or accurately identifying organic shorelines using aerial imagery and field reconnaissance data.

In order to deliver updated shoreline information to stakeholders and policy makers, we devised a manual image classification workflow, shown in Fig. 1, that adheres to the established protocols from previous mapping efforts^9,10^. Our chosen area of focus for implementing this methodology spanned 67 km of estuarine shoreline within the Neuse River Estuary (NRE), a significant tributary of the expansive Albemarle-Pamlico Estuarine System. The core components of this methodology encompassed automated digitization facilitated by a vertical datum-based indicator. Critical stages included rigorous quality assessment and meticulous manual editing, where the vertical datum-based indicator was thoughtfully overlaid with orthoimagery and Digital Elevation Models (DEMs). This overlay process served to facilitate thorough manual adjustments and error reduction. Furthermore, the accuracy of our classifications was validated through the utilization of oblique images captured from the water, ensuring a well-rounded and human-informed perspective. The ground truth data and classification maps developed for this study are available to the public for download at the University of North Carolina (UNC) Dataverse^13^. A separate database exists using the classified shoreline^13^ with LiDAR elevation data to identify areas of erosion and accretion between 2014 and 2020^14^.

## Methods

### Ground truth data

In the context of understanding alterations in the estuarine shoreline, it is imperative to identify, describe, and geolocate various landforms, habitats, and control structures along the shoreline. However, within the NRE, this task is often hindered by several factors, including dense vegetation, structures, and intricate lighting conditions. These complexities were underscored by Currin’s^11^ findings in the New River Estuary, a smaller estuary nearby to NRE, that found aerial data alone unsatisfactory for shoreline classification, necessitating the integration of ground truth data.

To address these challenges, field reconnaissance was conducted in the fall of 2020, covering approximately 67 km of the NRE shoreline via a small motorized watercraft. We employed ArcGIS Survey123 v.3.10 to record shoreline attributes and their corresponding XY coordinates, referenced to the World Geodetic Survey of 1984 and subsequently converted to the North American Datum of 1983 (2011) (NAD 83). These coordinates were then projected to the State Plane Coordinate System in meters. Simultaneously, oblique images were captured using a Sony R7R III camera. Photographic parameters such as shutter speed, aperture, and ISO were set to automatic, with a focus established at infinity. Each acquired image was complemented using a laser rangefinder and compass to measure the distance between the watercraft and the shoreline, along with the measurement angle. This allowed us to extrapolate the precise location of shoreline features from the vantage point of the watercraft. In total, the field reconnaissance resulted in a database comprising 395 distinct geographic features. Each entry in this repository includes geospatial coordinates and comprehensive attribute data relevant to the NRE shoreline as of 2020. This dataset, encompassing shoreline attributes and oblique imagery, is referred to as the ‘ground truth data’.

### LiDAR data

This study employed Light Detection and Ranging (LiDAR) Digital Elevation Models (DEMs) to identify slope gradients and landform contours used in the classification workflow. In the spring of 2020, an airborne LiDAR survey was flown over the NRE when waters were at or below normal levels (available at: (https://coast.noaa.gov/dataviewer/). The survey utilized a Riegl VQ880GII sensor. The vendor classified the LiDAR returns as ground and used them to generate LiDAR DEMs with horizontal resolutions dependent on the nominal pulse spacing (i.e., nominal pulse spacing multiplied by two). The 2020 LiDAR ground returns had a nominal pulse spacing of 0.5 m, which allowed for a 1 m resolution DEM to be created. The DEM was horizontally referenced to the NAD 83 (2011) using the State Plane Coordinate System, vertically referenced to the North American Vertical Datum of 1988 (hereinafter NAVD 88) using Geoid 18. All units were reported in meters.

### Aerial orthoimagery

To classify the state of the NRE shoreline as of 2020, high-resolution aerial imagery is needed. The best open source orthoimagery data of the NRE is available from the North Carolina Orthoimagery Program and the National Agriculture Imagery Program. Since 2012, the North Carolina Orthoimagery Program has produced 0.15 m horizontal resolution true-color orthoimages on a 4-year cycle. In addition to high-resolution imagery that does not exceed a horizontal RMSE of 0.45 m, another requirement of the Program is the collection of imagery during “leaf off” conditions. This allows for increasing the reliability of the 2020 shoreline classification because we now can see fine details such as bulkheads, riprap, and in many cases, bluff and sediment bank tops that could not be as easily identified from leaf-on imagery. We obtained county mosaics for Craven and Pamlico counties for 2016, and 2020 (available at: https://www.nconemap.gov/pages/imagery). Comparing the 2016 and 2020 imagery helped us distinguish between changes caused by human modifications and those resulting from natural factors like storms.

### Shoreline delineation and classification

The shoreline is generally defined as the physical interface of land and water^15^, yet this definition is challenging to objectively apply in the NRE due to wind-driven waves in a closed system where vegetation, buildings, and shadows also obscure the land and water boundary. Numerous shoreline indicators have been used in the literature to define shoreline change through time (for a review, see Boak & Turner^16^). In this study, the shoreline was determined through automated digitization using a vertical datum-based indicator rather than a visually discernable feature-based indicator to increase the objectiveness of our shoreline definition^16–18^. Specifically, we used the vertical control datum NAVD 88 (Geoid 18) to define the land–water boundary to minimize water level variation and shoreline obscurity between years. We used the Reclassify tool (3D Analyst Tools) in ArcGIS Pro v3.0 to remap all values less than zero as “wet” and all values greater than zero as “dry.” This output raster dataset was then converted to polyline features using the Raster to Polyline (Conversion) tool. Extraneous features in digitized lines can influence unfavorable orientations in which the shoreline is cast, so a smoothing method was used. We executed the Smooth Line (Cartography Tools) with the Polynomial Approximation with Exponential Kernel smoothing algorithm by Bodansky^19^. This algorithm’s effectiveness was visually confirmed by overlaying the smoothed shoreline results with the orthoimage. The smoothing tolerance of 10 m was chosen based on trial and error, topological errors were resolved, and the result was a line of the land-water boundary, ready for the subsequent manual image classification.

**Fig. 2:**
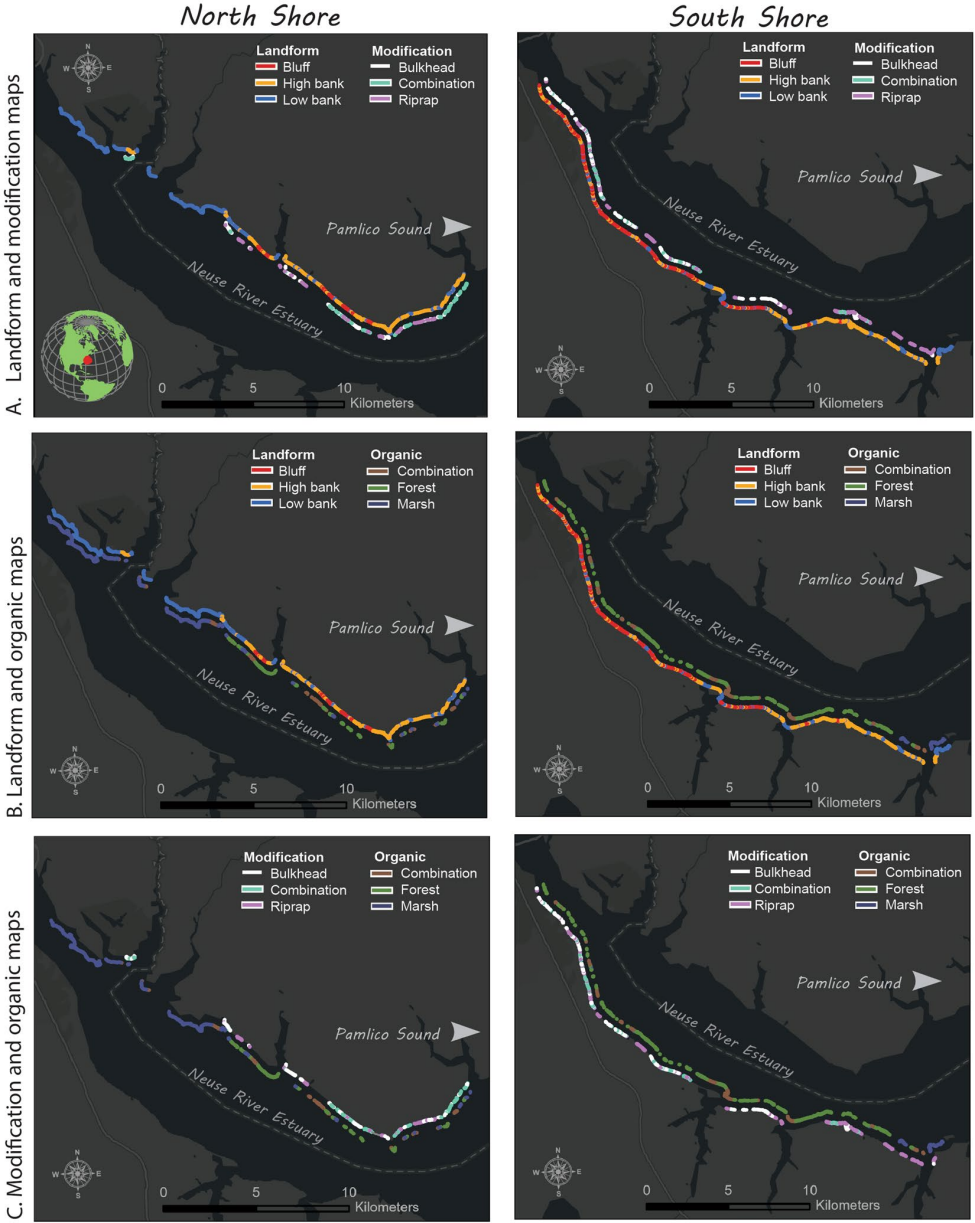
Maps of the north and south shores of the Neuse River Estuary, North Carolina, U.S., differentiated by orientation. Map (**A**) depicts landforms and shoreline modifications, (**B**) highlights landforms and organic shorelines, and (**C**) illustrates both modifications and organic shorelines in the Neuse River Estuary.

Using ArcGIS Pro v3.0, the 2020 shoreline feature was overlain with the respective orthoimagery, DEM, DEM-derived product from the Slope tool (3D Analyst Tools), and ground truth data viewed at an extent of 1:300 to 1:800 (see Fig. 1 above). At each ground truth location, the oblique image was also examined, which allowed us to field check our classification from a human’s eye view from the water. Five classification schemes were chosen for this study: A) sloped, B) landform, C) organic, D) modification, and E) remodified (refer to Fig. 1). Since most of the projects in the NRE currently focus on slope stabilization by reworking eroding bluffs into a gentler and more stable slope configuration using <30% slope gradient^20^, we used this threshold where all DEM grid cell slopes <30% were categorized as “Yes” indicating the landform was stably sloped. Landforms were identified from the DEMs as low bank (0 to 1.5 m in elevation), high bank (1.6 to 6 m), and bluff (>6.1 m) following the classification presented by Riggs & Ames^21^. Manual inspection showed that these thresholds represented the landforms in the NRE well. Organic shorelines were defined as those with marsh, forest^21^, or a combination. The modification types found in the ground truth data included riprap, bulkhead, and combination (e.g., riprap and bulkhead). Areas that represented change by human activity (e.g., re-modification) between 2016 and 2020 were discerned from those that experienced other changes due to the environment (e.g., Hurricane Florence in 2018). The result was a classified 2020 shoreline segmented and assigned each of the five shoreline classification categories shown in Fig. 1. An example of maps showing the features is shown in Fig. 2.

## Data Records

The ground truth data and classification maps are readily accessible for download in a convenient zip-archive format via the UNC Dataverse data repository^13^, with a modest file size of 2.5GB. This dataset comprises three primary components: 1) field photos, 2) field GIS data, and 3) shoreline classifications. The first component encompasses a collection of 395 oblique images stored in.jpg format. These images adhere to a systematic naming convention, employing the format “SurveyDay_ImageNumber.” For example, ‘Survey1_001’ denotes a photo captured during Survey Day 1, with the image number 001. The second component includes related geospatial coordinates and attribute data provided as point shapefile and feature class datasets named “Neuse2020Survey.” Within these datasets, each of the 395 points symbolizes the precise geographic location of a field photo. Importantly, the ‘ImageNumber’ attribute corresponds to the image labelling adopted for the field photos. Thus, ‘ImageNumber 100’ is in perfect correspondence with ‘Survey1_100,’ simplifying data interpretation and streamlining searches between datasets. The final component includes the 2020 shoreline classification, stored as polyline shapefiles and feature class datasets labelled ‘NRE_Northshore_2020_Classification’ and ‘NRE_Southshore_2020_Classification.’ These datasets contain attributes that facilitate the identification of various landforms, organic shorelines, modifications, and regrading (stably sloped areas).

Notably, all geographic data in this dataset adhere to the horizontal referencing standards of the NAD 83 (2011) while employing the State Plane Coordinate System. Furthermore, the units employed within this dataset are standardized to meters to promote clarity and uniformity. These meticulous details ensure the dataset’s precision and accessibility for diverse applications in research and analysis.

## Technical Validation

A quality assessment and quality control (QA/QC) was performed by manual editing, where time was broken into 30-minute intervals with small breaks in between to help reduce user error. QA/QC procedures included checking for incorrect information and revisiting the ancillary data several times. The classified shoreline was overlain with the ancillary data of orthoimagery, DEM, slope, and point file of the 395 oblique image locations at a viewing extent of 1:800. At each of the 395 points, the oblique image was also viewed on screen. Shoreline segments lacking ground truth data that also contain an obscure shoreline were flagged, e.g., due to vegetation, and, when in doubt, were removed from further analysis, resulting in ~250 m of shoreline removed^22–24^.

## Usage Notes

Classified shoreline data, encompassing details about distinct land features, natural shoreline elements, and erosion control structures, offers many possibilities for guiding research, management, and decision-making in coastal regions. Some valuable applications for this type of data include, but are not limited to, the following:

- Coastal Erosion Analysis: The data can be used to assess the vulnerability of different shoreline areas to shoreline recession and erosion^7,11,18,22^. Identifying the location and types of shoreline modification procedures (e.g., bulkhead, riprap) allows researchers to evaluate their effectiveness in reducing erosion^11^.
- Coastal Ecosystem Assessment: By examining how various landforms and organic shoreline types are distributed across the landscape, we gain insights into how human alterations can impact the preservation or degradation of threatened estuarine habitats^23–25^.
- Coastal Management: Agencies and non-profits that manage the coast often lack up-to-date and reliable information about the shoreline^9–11,26,27^. To minimize environmental impacts, shoreline classification data are crucial to facilitating and encouraging green shorelines^27^.
- Community Outreach: The data can raise the local communities’ awareness of how humans modify the shoreline and how these modifications impact the ecosystem. It can be used to create communication/visualization tools suitable to a general audience to enhance stakeholder engagement^25,27,28^.

## Data Availability

No custom code was used in the generation or processing of this dataset. Software used in this study includes ArcGIS Pro v3.0 and ArcGIS Survey123 v.3.10.

## References

[1] Barbier EB (2011). The value of estuarine and coastal ecosystem services. Ecol. Monogr..

[2] Dalrymple RW, Zaitlin BA, Boyd R (1992). Estuarine facies models: conceptual basis and stratigraphic implications. J. Sediment. Petrol..

[3] Laird, M.P., Theberge, B.L., & Jones, N.B. *An Assessment of Estuarine and Nearshore Marine Environments*. Special Reports in Applied Marine Science and Ocean Engineering (SRAMSOE) No. 93. Virginia Institute of Marine Science, William & Mary. 10.21220/V5F161 (1975).

[4] Costanza R (1997). The value of the world’s ecosystem services and natural capital. Nature..

[5] Lotze HK (2006). Depletion, degradation, and recovery potential of estuaries and coastal seas. Science..

[6] Kennish, M. J. Anthropogenic Drivers of Estuarine Change. *Climate Change and Estuaries*. 75–98 CRC Press, (2024).

[7] Phillips JD (2022). Geomorphic impacts of Hurricane Florence on the lower Neuse River: Portents and particulars. Geomorphology..

[8] Paerl HW (2019). Recent increase in catastrophic tropical cyclone flooding in coastal North Carolina, USA: Long-term observations suggest a regime shift. Sci Rep..

[9] Geis, S. & Bendell, B. *Charting the Estuarine Environment: A Methodology Spatially Delineating a Contiguous, Estuarine Shoreline of North Carolina*. NC Division of Coastal Management. https://files.nc.gov/ncdeq/Coastal%20Management/GIS/Data/ESMP-20100115-Charting-the-Estuarine-Environment.pdf (2010).

[10] McVerry, K. *North Carolina estuarine shoreline mapping project, statewide and county statistics*. North Carolina Division of Coastal Management. https://www.deq.nc.gov/documents/pdf/esmp-analysis-report-final-20130117/download (2012).

[11] Currin C, Davis J, Baron LC, Malhotra A (2015). Shoreline Change in the New River Estuary, North Carolina: rates and consequences. J. Coast. Res..

[12] Blaschke T (2010). Object based image analysis for remote sensing. ISPRS J. Photogramm. Remote Sens..

[13] Sirianni H, Richter J, Sirianni M, Pettyjohn S (2023). UNC Dataverse.

[14] Sirianni H, Sarah P, Sirianni M (2023). UNC Dataverse.

[15] Stockdon HF, Sallenger AH, List JH, Holman RA (2002). Estimation of shoreline position and change using airborne topographic lidar data. J. Coast. Res..

[16] Boak EH, Turner IL (2005). Shoreline definition and detection: a review. J. Coast. Res..

[17] Gens R (2010). Remote sensing of coastlines: Detection, extraction and monitoring. Int. J. Remote Sens..

[18] Sirianni H (2022). Quantifying recent storm-induced change on a small fetch-limited barrier island along North Carolina’s Crystal Coast using aerial imagery and LiDAR. Coasts..

[19] Bodansky, E., Gribov, A. & Pilouk, M. Smoothing and Compression of Lines Obtained by Raster-to-Vector Conversion. LNCS 2390, Springer. 256–265 (2002).

[20] Herring, B. Post-Florence improvements still in the works thanks to disaster recovery funds secured by NCDA & CS. *In The Field Blog*. https://blog.ncagr.gov/2021/03/17/post-florence-improvements-still-in-the-works-thanks-to-disaster-recovery-funds-secured-by-ncdacs/ (2021).

[21] Riggs, S.R & Ames, D.V. *Drowning the North Carolina Coast: Sea-level Rise and Estuarine Dynamics*. UNC-SG-03-04. North Carolina Department of Environment and Natural Resources and North Carolina Sea Grant. Raleigh, NC. (2003).

[22] Eulie DO, Walsh JP, Corbett DR, Mulligan RP (2017). Temporal and Spatial Dynamics of Estuarine Shoreline Change in the Albemarle-Pamlico Estuarine System, North Carolina, USA. Estuaries Coast..

[23] Gittman R (2016). Living shorelines can enhance the nursery role of threatened estuarine habitats. Ecol. Appl..

[24] Smith CS (2017). Hurricane damage along natural and hardened estuarine shorelines: Using homeowner experiences to promote nature-based coastal protection. Mar. Policy.

[25] Polk, M. A., & Eulie, D. O. Effectiveness of living shorelines as an erosion control method in North Carolina. *Estuaries and Coasts*, **41**(8) (2018).

[26] Correll-Brown R (2022). Shifting baselines may undermine shoreline management efforts in the United States. Front. clim.

[27] Luscher, A. Maryland shorelines on-line: a web portal and geospatial tool for shoreline planning and management in Maryland. Management, Policy, Science and Engineering of Nonstructural Erosion Control in the Chesapeake Bay: Proceedings of the 2006 Living Shoreline Summit. Editor Sandra Y. Erdle. CRC Publ. No. 08-164, Gloucester Point, VA 136pp (2008).

[28] DeCock-Caspell M (2021). & and Liette Vasseur. Visualizations as a tool to increase community engagement in climate change adaptation decision-making. FACETS..

